# A Study Protocol for Testing the Effectiveness of User-Generated Content in Reducing Excessive Consumption

**DOI:** 10.3389/fpsyg.2017.00972

**Published:** 2017-06-09

**Authors:** Atar Herziger, Amel Benzerga, Jana Berkessel, Niken L. Dinartika, Matija Franklin, Kamilla K. Steinnes, Felicia Sundström

**Affiliations:** ^1^Cologne Graduate School in Management, Economics and Social Sciences, University of CologneCologne, Germany; ^2^Division of Psychology and Language Sciences, University College LondonLondon, United Kingdom; ^3^Department of Psychology, University of CologneCologne, Germany; ^4^Department of Psychology, Maastricht UniversityMaastricht, Netherlands; ^5^Department of Psychology, University of CambridgeCambridge, United Kingdom; ^6^Department of Psychology, University of OsloOslo, Norway; ^7^Department of Psychology, Uppsala UniversityUppsala, Sweden

**Keywords:** voluntary simplicity, ethical consumption, intervention studies, user-generated content, excessive consumption, consumer values, self-enhancement, self-transcendence

## Abstract

Excessive consumption is on the rise, as is apparent in growing financial debt and global greenhouse gas emissions. Voluntary simplicity, a lifestyle choice of reduced consumption and sustainable consumer behavior, provides a potential solution for excessive consumers. However, voluntary simplicity is unpopular, difficult to adopt, and under researched. The outlined research project will test a method of promoting voluntary simplicity via user-generated content, thus mimicking an existing social media trend (Minimalism) in an empirical research design. The project will test (a) whether the Minimalism trend could benefit consumers interested in reducing their consumption, and (b) whether self-transcendence (i.e., biospheric) and self-enhancement (i.e., egoistic and hedonic) values and goals have a similar impact in promoting voluntary simplicity. A one-week intervention program will test the efficacy of watching user-generated voluntary simplicity videos in reducing non-essential consumption. Each of the two intervention conditions will present participants with similar tutorial videos on consumption reduction (e.g., decluttering, donating), while priming the relevant values and goals (self-transcendence or self-enhancement). These interventions will be compared to a control condition, involving no user-generated content. Participants will undergo baseline and post-intervention evaluations of: voluntary simplicity attitudes and behaviors, buying and shopping behaviors, values and goals in reducing consumption, and life satisfaction. Experience sampling will monitor affective state during the intervention. We provide a detailed stepwise procedure, materials, and equipment necessary for executing this intervention. The outlined research design is expected to contribute to the limited literature on voluntary simplicity, online behavioral change interventions, and the use of social marketing principles in consumer interventions.

## Excessive consumption

Consumption becomes excessive when it exhausts the consumer's financial or mental resources, or when it creates extreme environmental consequences, thus negatively affecting individual and societal well-being (Sheth et al., 2011). During the last decade, US consumption has risen dramatically; in 2007, consumption accounted for 72% of the American GDP. One factor which may contribute to excessive consumption is Materialism. Materialistic individuals consider material goods as an important part of their identity (Belk, 1984). They perceive material possessions as central to their status and happiness (Richins and Dawson, 1992). In a recent meta-analysis examining materialism and personal well-being, a significant negative relationship was found between the two (Dittmar et al., 2014). In an effort to reduce excessive consumption and increase consumer well-being, this project proposes a method to engage consumers in a reduced consumption lifestyle, namely, voluntary simplicity.

## Voluntary simplicity

A frequently proposed solution to excessive consumption and its negative affects is voluntary simplicity (Etzioni, 1998; Alexander and Ussher, 2012); the consumer movement of adopting a simplistic lifestyle. Voluntary simplicity involves a consumer's independent decision to reduce and replace non-essential products and services with non-material life elements, in an attempt to increase life satisfaction and meaning (Etzioni, 1998; Huneke, 2005). Non-essential items and services can be defined as things that are irrelevant for the achievement of one's life purpose (McGouran and Prothero, 2016). For example, voluntary simplicity behaviors include: composting, recycling, decluttering (Huneke, 2005), self-sufficiency and use of pro-environmental transportation (Alexander and Ussher, 2012). While this lifestyle is promising in its potential to reduce consumption and increase well-being, is it difficult to adopt (McGouran and Prothero, 2016).

### Minimalism

The scholarly concept of voluntary simplicity has recently been adapted into popular culture, under the term *Minimalism*. Minimalism often encompasses user-generated content, such as videos, texts, and images that promote consumption reduction. Minimalism content is largely shared via YouTube, Facebook, Twitter and similar online social networking services. Additionally, there are numerous blogs, online forums and websites devoted to Minimalism^1^. In these online sources, viewers can find information about Minimalism, tips on how to adopt this lifestyle, tutorials, and personal narratives of successful minimalists.

The Minimalism phenomenon, to a lesser degree than voluntary simplicity, seems to utilize an aesthetic approach. Minimalism can be studied as an aesthetically pleasing trend, relating to both fashion and web design. For example, narratives of fashion bloggers dedicated to a Minimalistic style revealed a strong emphasis on simplicity, elegance, sophistication and cleanliness, which were achieved in particular by choice of coloring and scarce use of patterns (Karg, 2015). It is yet early to determine whether the Minimalism phenomenon is a passing trend or a new wave of conscious consumerism, but Minimalism may have the power to engage consumers in sustainable behaviors.

## Self-transcendence and self-enhancement values and goals

One way through which consumer interventions could reduce materialism and promote sustainable consumption, is through the activation of relevant values and goals (Kasser, 2016). In other words, Minimalism videos may not only serve the purpose of directly increasing voluntary simplicity behaviors through tutorials; these videos are also a channel through which relevant ecological values could be activated and strengthened. Thus, user-generated content on Minimalism could promote personal norms of sustainable consumption, creating longer-term impact on consumer behavior.

Previous research has consistently shown that a biospheric value orientation is positively associated with ecological attitudes and beliefs, and is thus conducive to sustainable behavior (e.g., Stern, 2000; de Groot and Steg, 2008). A biospheric value orientation is a type of self-transcendence value, which emphasizes the environment and the biosphere over one's own personal benefit (Stern et al., 1993).

Self-enhancement values, on the other hand, promote one's own benefit over that of others or the environment, e.g., egoistic and hedonic values (Schwartz, 1992). Egoistic and hedonic values are negatively associated with ecological attitudes and sustainable behaviors (Steg et al., 2014). Moreover, these values are closely clustered with materialism, and thus could promote excessive consumption (Burroughs and Rindfleisch, 2002; Kasser, 2016).

### Self-transcendence and self-enhancement in minimalism videos

Although, previous literature suggests that self-enhancement values have a negative impact on sustainable behavior, user-generated content on Minimalism seems to be focused exactly on these values. Exploratory research of the Minimalism phenomenon found that engagement in Minimalism is associated with a personal goal to reduce stress (hedonism) and save money (egoism), but not with environmental concern (Herziger, unpublished manuscript). Thus, we argue that self-enhancement goals might be utilized to promote sustainable consumer behavior. However, it remains unclear whether self-enhancement goals could be as effective as self-transcendence goals in promoting sustainable behavior.

## Objectives

The project will test whether user-generated content promoting either self-transcendence or self-enhancement values and goals could be effective in reducing excessive consumption, as well as increasing well-being. The effectiveness of the interventions will be tested by measuring their effects on life-satisfaction, intent to adopt voluntary simplicity, and purchasing intent and behavior. The project's main research questions are:

1. Could user-generated content promoting voluntary simplicity reduce excessive consumption?
2. Are self-enhancement goals as effective in promoting sustainable behavior as self-transcendence goals, in user-generated Minimalism content?

We are also interested in whether exposure to user-generated content promoting voluntary simplicity affects one's well-being. Finally, the relationship between one's intent to reduce consumption and one's consumption behavior might generate meaningful insights.

## Design

A weeklong intervention program will test the efficacy of watching voluntary simplicity videos in reducing non-essential consumption. The first condition will promote voluntary simplicity while priming self-transcendence values and goals, and the second condition will promote voluntary simplicity while priming self-enhancement values and goals. These two interventions will be compared with a control condition which only measures participant's affective state during the intervention week. All participants will undergo baseline and post-intervention evaluations of values and goals in consumption reduction, voluntary simplicity attitudes and behaviors, spending behaviors, and life satisfaction directly before and directly following the intervention, respectively. A one-month follow up measurement will also be taken. The difference between measurements before and after the intervention will serve as our dependent variables.

## Intervention

A key component in this study is the video stimuli employed. Videos have proven to be more effective and persuasive than other modalities of communication (Mohammadi et al., 2013). To maintain a high level of reliability and validity, the research team will create the intervention videos in-house. To create valid video stimuli several measures will be taken:

### Video checklists

Video checklists describe the key concepts and behaviors to be addressed in the video stimuli. These checklists detail the following stimuli requirements, per condition:

1. Content: information conveyed (stable across intervention conditions)
2. Condition priming: what values and goals will be primed in the opening and closing statements of the videos, as well as the visual stimuli (self-transcendence vs. self-enhancement)
3. Style: the presenter's tone and expression (stable across intervention conditions).

Due to internal reliability concerns, one presenter will appear in all videos, for both intervention conditions. The presenter will be a female in her twenties, similarly to many Minimalism vloggers. Appendix A presents an example of a video checklist.

The research team will contact several social and consumer psychologists to review these checklists and approve that they (a) prime self-transcendence and self-enhancement values and goals, per condition, and (b) promote consumption reduction, prior to video creation.

### Video pre-testing

The videos will be pretested in a convenience sample of international psychology students at the advanced undergraduate and graduate level. We aim to test the face validity of the videos. Thus, the pre-test population will consist of young consumers with a basic scientific knowledge on motivation, values, and goals. The pre-test sample will therefore be slightly different from the population addressed in the main study. Participants will watch subsets of videos from both intervention conditions and rate the face validity of the videos (e.g., “Did the presenter promote biospheric values?”, “Did the presenter promote egoistic values?”). They will also note any manipulation concerns or potential confounds in the video stimuli of both conditions. Additionally, participants will rate how entertained, focused and interested they were when watching the videos. The latter measures will be used to estimate main-study participants' motivation in watching the daily video stimuli during the weeklong intervention. If a video receives a low score on either validity or entertainment value during the pre-test, the research team will revise the videos based on pre-test comments, and re-test them on a similar sample.

### Mobile application

A mobile application will be developed and used in the study for stimuli presentation, data collection, communication, and once the study is complete, a personalized progress report. Video stimuli will be embedded into Qualtrics questionnaires, which will be linked in the mobile application. This will allow participants to easily fill-out the questionnaires before and after viewing the video stimuli. Time spent viewing the videos will be recorded by a “timing” function in Qualtrics.

The mobile application push-notification system will remind participants to view the video and fill in a questionnaire on a daily basis throughout the weeklong intervention. Thus, participants will receive one push-notification a day, until the intervention is complete. The mobile application will also allow both the participants and research team to communicate via email. This will be useful if any problems or questions arise. Finally, users of the mobile application will receive a personalized progress report once the study is completed.

## Procedure

**Figure 1:**
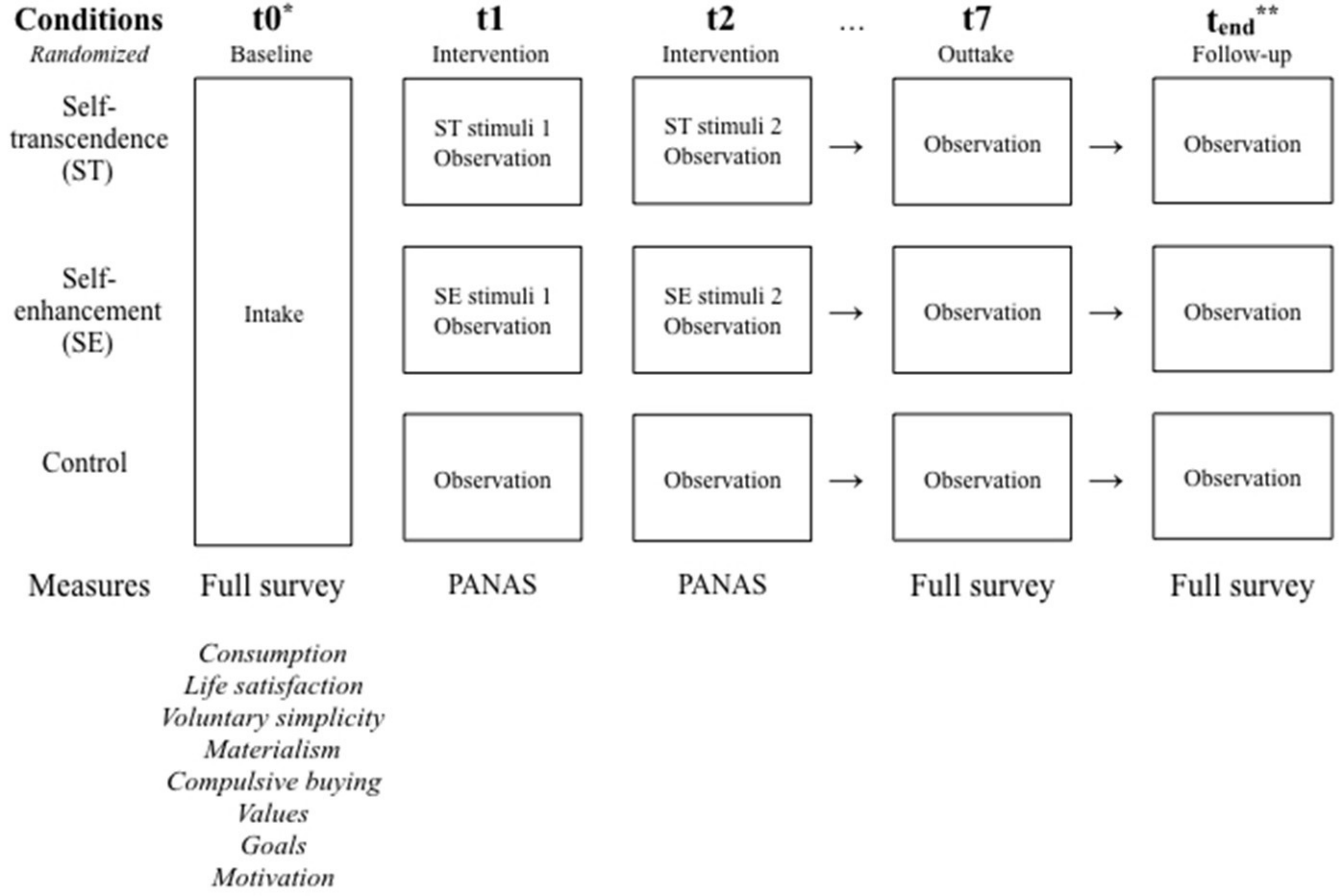
Study outline. ^*^t+1 equals one day; t0−t7 are completed within one week. ^**^t_end_ will take place one month after intervention completion (t7).

At baseline, all participants will download a free mobile application. Participants will complete an intake survey on Qualtrics, through a link in the mobile application. Following intake, participants will be randomly allocated to one of the three experimental conditions, and complete a battery of questionnaires measuring dependent and independent variables (see t0 in Figure 1). After baseline measurements, the intervention will commence. In the two intervention conditions, participants will watch one video daily for 6 days. Both intervention and control condition participants will complete a daily mood measurement (see t1 through t6 in Figure 1). On the last day of the intervention, participants will complete questionnaires measuring the dependent and independent variables a second time (see t7 in Figure 1). One month after intervention completion, the dependent variables will be measured again (see t_end_ in Figure 1).

## Methods

### Ethics process

Due to the study's intervention design, careful consideration was put into ethical procedures executed by the research team. Ethical approval has been granted for this study. In addition to standard procedures, such as voluntary participation and dropout, several processes are in place to reduce potential harm to participants' well-being.

Firstly, participants who struggle with compulsive buying, hoarding, or other addictive behaviors are regarded as sensitive populations, and will be asked through a consent form not to take part in the study. Individuals diagnosed with Compulsive Buying Disorder (CBD) repeatedly engage in excessive spending behaviors and cognitions due to irrepressible impulses, which result in anxiety or impairment (Black, 2007). CBD diagnosed individuals will be ineligible to participate in the study because of two main reasons; (a) the topic and nature of the study could potentially cause harm or distress to the individual and (b) the study's purpose, to explore potential solutions to reduce excessive consumption, will be directed toward the general population of consumers and not those suffering from a clinical buying disorder. Thus, an in-take survey will employ a validated self-report clinical screener for CBD (Faber and O'Guinn, 1992). Participants scoring over the clinical threshold will be excluded from the study; they will be notified of their ineligibility, via email, within two days of their in-take questionnaire submission. They will also be sent additional information on professional help networks for compulsive buyers^2^. A diagnosis will not be provided to participants due to the research team's lack of clinical training and the absence of differential diagnosis.

Similarly, participants will be asked to report any undesirable changes in mood and behavior during the intervention by contacting the research team. Participants reporting an increase in buying behavior or severe decline in well-being, will be interviewed by e-mail, and dismissed from the study. Full reporting of these instances, should there be any, will be included in the results section of disseminated work emerging from this project.

After the intervention is completed, including a one-month follow-up, the research team will conduct initial analysis on the effectiveness of the employed interventions. Specifically, conducted analysis will test whether either one or two of the employed interventions prove to be beneficial in reducing excessive consumption and increasing well-being, in comparison to the control condition. If one or both interventions prove to be effective, they will be offered to participants who did not experience them while participating in the study. Thus, all participants will be afforded the opportunity to take part in an effective intervention for consumption reduction, should the research team identify one. Participants will also receive debriefing of the study and will be exposed to all three conditions employed in the study design.

### Participant outreach

#### Sample size

Two hundred and fifty English-speaking subjects will be recruited for this study globally, via online methods. The number of participants needed is based on an a priori power analysis using G^*^Power software (*F*-test, ANOVA: Repeated measures, within-between-interaction; 1− β = .80, α = .05, Cohen's f = .10), which revealed that the study would require a total sample size of *N* = 198 (Faul et al., 2007). An additional 52 participants have been added to this estimated sample size to account for an assumed 20% dropout rate.

#### Participant criteria

Participants will sign a consent form electronically stating that; (a) they are at least 18 years of age or of legal age in their country of residence, (b) they have not been clinically diagnosed with a disorder related to impulse control or addictions, (c) they express both a desire and difficulty to reduce their non-essential consumption, (d) they have not previously followed the social media trend of consumer Minimalism.

#### Recruitment methods

Participants will be recruited through convenience sampling via two main avenues; (a) social media posts in consumption related forums and sites as well as Facebook pages and groups, and (b) campus posters circulated around the universities of the research team. The main appeal to participants in all avenues will follow a “question and proposal” format, i.e., “Do you want to decrease your buying? Sign-up to try out an experimental treatment!” Each poster and online recruitment posting will feature a QR code that participants can scan with their smartphones to download the intervention mobile application. The consent form will be embedded in the in-take questionnaire.

#### Participant commitment through feedback

Efforts to promote continued participation will be aimed at both extrinsic and intrinsic motivation, with a primary emphasis on the latter. Two intrinsic, motivational aspects will be addressed in the recruitment call; (a) participants will be offered to try treatments that may help them with their reported, personal goal of reducing non-essential material consumption and (b) participants will receive feedback on their personal progress following the intervention.

To further motivate participants to remain in the study throughout the weeklong intervention, a lottery prize of 100 euros will be raffled to one participant who completed the full intervention. The winning participant will be able to receive this prize via either bank transfer or a donation made on their behalf to a charity of their choice.

### Surveys and intervention

After baseline measurement is completed, participants in each intervention group will be requested to watch one video per day for six days, and answer a short questionnaire following each video. The videos will be between three and five minutes long and consist of a young, female presenter, talking to the camera as if talking to an audience.

The main content of the videos in both intervention conditions will be identical. However, the opening and closing sections of each video will be filmed separately, and emphasize either self-transcendence or self-enhancement values and goals, per condition. For example, in the self-transcendence condition the presenter will say, “We are here because we want to *make the world a better place*. By being *environmentally friendly, conscience* and *ethical*, we can *make a positive impact on the world*.” Conversely, in the self-enhancement condition, the presenter will say, “We are here because we want to *make our lives better*. By being *less stressed, more in control*, and *spending wisely*, we can *make a positive impact on our own lives*.” Moreover, visual stimuli integrated into the videos will prime participants of either self-transcendence, biospheric values (e.g., gardens, lakes, plants) or self-enhancement, hedonic and egoistic values (e.g., friends chatting and drinking coffee together). In the control condition, there will be no intervention, but participants will be pinged by the mobile application to answer a survey once per day for six days. Follow-up measurements will be conducted via web-site one month after the last intervention day for all three groups.

## Materials and equipment

### Smartphone application

The smartphone application will contain four key features:

1. Questionnaires: the mobile application will link participants to Qualtrics questionnaires, including video stimuli. These will be presented to the participants at in-take, during the daily intervention, and at post-intervention assessment;
2. Notifications: a push-notification system will remind participants to view the stimuli and complete the questionnaires every day, while the intervention is in progress;
3. Communication with the research team: an email link will allow participants to contact the research team directly;
4. Personalized progress reports: participants will receive a link to a report of their post-intervention progress once the study is complete.

### Measures

#### Demographic questionnaire

Participants will be asked to provide demographic information, such as gender, native language, education level, age, ethnicity, and country of residence.

#### Intake screeners

##### Assessment of Compulsive Buying Behavior

The Clinical Screener (CBCS; Faber and O'Guinn, 1992) is one of the most validated and replicable self-report scales measuring compulsive buying behavior, having been found to correctly classify 88% of compulsive buyers (Faber and O'Guinn, 1992). The CBCS contains seven statements that respondents are required to rate their agreement with (1 = Strongly disagree to 5 = Strongly agree) (e.g., “If I have any money left at the end of the pay period, I just have to spend it”) or their frequency of experiencing given behaviors/feelings (1 = very often to 5 = never) (e.g., “Bought things even though I could not afford them”) on five-point Likert scales. A total score is calculated, and a threshold measurement determines whether an individual is considered a compulsive buyer.

Faber and O'Guinn's (1992) compulsive buying scale has, however, been criticized for only addressing the impulse control dimension of compulsive buying behavior and not the obsessive-compulsive dimension, as well as not being accommodating to buyers that have higher incomes (Ridgway et al., 2008). To compensate for these limitations, the Compulsive-Buying Index (CBI) developed by Ridgway et al. (2008) will be used in addition to the CBCS, thus presenting a more comprehensive measurement of compulsive buying behavior.

The Compulsive-Buying Index (CBI) is a well-validated measure of compulsive buying behavior (Ridgway et al., 2008). The CBI contains six items measuring two dimensions of compulsive buying; three items for obsessive-compulsive buying (e.g., “My closet has unopened shopping bags in it”) and three items for impulsive buying (e.g., “I buy things I don't need”). Responses are given on a seven-point Likert scale either indicating agreement with statements (1 = strongly disagree to 7 = strongly agree) or frequency of experiencing given behaviors/feelings (1 = never to 7 = very often). A total score is calculated, and a threshold measurement determines whether an individual is considered a compulsive buyer.

##### Assessment of Voluntary Simplicity

*Lifestyles*. The Voluntary Simplicity Lifestyles scale used in this study (VSL; Nepomuceno and Laroche, 2015) is an adapted version of Iwata's original voluntary simplicity scale (Iwata, 1997), a validated self-reported measure of the adoption of the voluntary simplicity consumer lifestyle. This adapted scale is made up of nine statements describing a number of behaviors that are consistent with a voluntary simplistic lifestyle, such as the scale item “I fully adhere to a simple lifestyle and only buy necessities.” Participants are required to rate their agreement with these statements on a five-point Likert scale (1 = definitely disagree to 5 = definitely agree), indicating the degree to which they engage in the suggested simplistic lifestyle choices.

*Involvement*. The Personal Involvement Inventory (PII; Zaichkowsky, 1994) is considered a validated context-free measure of one's motivation to be involved with certain concepts, behaviors or products. This study tailored the PII to measure consumers' voluntary simplicity involvement. Involvement is measured on a self-report semantic scale made up of 10 items. The scale can be divided into two subscales: the affective subscale (e.g., interesting, appealing) and the cognitive subscale (e.g., important, relevant). Participants will be asked to judge voluntary simplicity across all cognitive and affective adjectives. Participants rate each item along a bipolar adjective scale (e.g., “interesting” or “boring”), judging how close they deem voluntary simplicity to either opposing adjective. Responses are given on a seven-point scale and six items require reverse scoring. A total involvement score is calculated where higher scores represent higher involvement.

##### Assessment of Materialism

The Materialism Scale (MS; Richins and Dawson, 1992) is a validated self-report scale measuring materialism value. The 18-item scale measures three dimensions of materialism; success, centrality and happiness, which describe different motivations for acquiring material possessions. Six items measure success, indicating the perception that possessions are indicators of life-success (e.g., “I admire people who own expensive homes, cars, and clothes”). Seven items measure centrality, representing the general importance of acquisition and possession (e.g., “I enjoy spending money on things that aren't practical”). The last five items measure happiness, representing the perception that possession is necessary for happiness (e.g., “My life would be better if I owned certain things I don't have”). The MS is measured on a five-point Likert scale (1 = strongly disagree, 5 = strongly agree) and eight items require reverse coding. An overall scale score is calculated from the three dimensions, a higher score suggesting higher materialism than a lower score.

##### Assessment of Egoistic, Altruistic, and Biospheric Values Orientations

The Value Instrument is an adapted, validated measure of egoistic, altruistic and biospheric value orientations (de Groot and Steg, 2008). This scale is an adapted form of the Schwartz value scale (Schwartz, 1992), and consists of 13 items: five for egoistic (e.g., wealth), four for altruistic (e.g., equality), and four for biospheric (e.g., unity with nature) value orientations. Internal reliabilities for these sub-scales range from .73 to .86. As in the Schwartz value scale, participants rate the importance of the 13 items “as a guiding principle in their lives” on a nine point Likert-scale (–1 = opposed to my values, 0 = not important to 7 = extremely important). In the instructions segment, respondents are requested to vary the scores they provide each value and only rate very few of them as extremely important.

##### Assessment of Satisfaction with Life

The Satisfaction with Life Scale (SWLS; Diener et al., 1985) is a validated measure of subjective well-being, characterized by favorable psychometric properties, such as convergent validity with other similar measures, cross-cultural validity, and temporal stability (Pavot and Diener, 2008). The five-item self-report scale measures global life satisfaction by scoring participant agreement to a statement such as “I am satisfied with my life” on a seven point Likert-scale (1 = strongly disagree to 7 = strongly agree). A total score can range between five (low satisfaction) and 35 (high satisfaction) suggesting the degree of life satisfaction of the participant.

##### Assessment of Motivation

Participants will be asked to rate their motivation source and strength for taking part in the study. Motivation sources will match the aforementioned value orientations (e.g., egoistic and hedonic: “improve my well-being,” “save my money”; altruistic: “improve the lives of others around me,” “donate more to others”; and biospheric: “improve the future of the environment,” “reduce my carbon footprint”). Participants will rate their agreement with these motivations similarly to the value instrument (–1 = opposed to my motivation, 0 = not motivating to 7 = extremely motivating).

##### Assessment of Consumption Related Attitudes and Behaviors

*Shopping intention*. Shopping intention will be measured by adapting the three items of the eight-point semantic scale of behavioral buying intention (Baker and Churchill, 1977). The original items were intended to measure participants' intention to buy a specific product. In this study, the items will be adapted to suit the context of the non-essential product buying intention. Participants will be asked to think of a type of product they would normally buy, which is non-essential (e.g., clothing, sweet snacks, coffee). Then, they will be asked about their purchase intent for this product, for example: “Would you buy this non-essential product within the next seven days?” In response to the question, participants should indicate their intention on an eight-point semantic scale of “yes—definitely” to “no—definitely not.”

*Buying frequency and behavior*. Two questionnaires (see Appendix B) measuring shopping frequency (Section A, seven items) and average weekly non-essential expenditures (Section B, two items) will be administered to participants. The order of the items within both sections will be randomized. Responses for five of the total nine items of the two questionnaires will be given on a six-point Likert scale, ranging from zero to five (e.g., “How likely are you to shop for something you need in the coming week?”) that will be averaged within each section. The remaining four items will require an open response by the participant (e.g., “How many times do you typically go shopping in a week?”).

#### Intervention questionnaire

##### Short Mood Scale (PANAS).

The Short Form Positive and Negative Affect Schedule (I-PANAS-SF; Thompson, 2007) is a short version of the widely-used measure of the variation in affect, the Positive and Negative Affect Schedule (PANAS; Watson et al., 1988). PANAS is a well-validated measure of affect with strong psychometric properties (Watson et al., 1988; Crawford and Henry, 2004), demonstrated cross-culturally (e.g., Joiner et al., 1997) and within both community and clinical samples (Leue and Beauducel, 2011). The I-PANAS-SF is a 10-item self-report scale that is divided into two subscales, Negative Affect (NA; afraid, ashamed, hostile, nervous, and upset) and Positive Affect (PA; active, alert, attentive, determined, and inspired). Participants are required to judge the extent to which they experience the NA and PA adjectives on a seven-point Likert scale (0 = never to 7 = always) in response to the statement: “Thinking about yourself and how you normally feel, to what extent do you generally feel.” This phrasing will be revised to address the current moment.

#### Post-intervention questionnaire

The effectiveness of the intervention will be measured by the difference of participants' pre- and post-intervention measurements, controlling for covariates. Therefore, all intake questionnaires will be repeated in the post-intervention phase. Along with these repeated scales, several additional measures will be administered.

Two additional questions will measure participants' attitude toward the videos. The first item will measure whether participants would recommend the video to friends with a similar goal (i.e., “Would you recommend these videos to a friend who also wanted to reduce their non-essential consumption?”). The second item will measure participants' intention to continue watching similar videos online (i.e., “Would you watch similar videos online?”). Participants would be required to indicate their answer on a nine-point scale (0 = not at all to 8 = definitely) for both questions. Lastly, an open-ended question will allow participants to share what they thought was interesting, difficult, or helpful during the one week of intervention.

## Proposed analysis

To test the hypotheses, statistical software including Excel, SPSS, and R will be utilized.

### Consumption reduction—behavior

**Figure 2:**
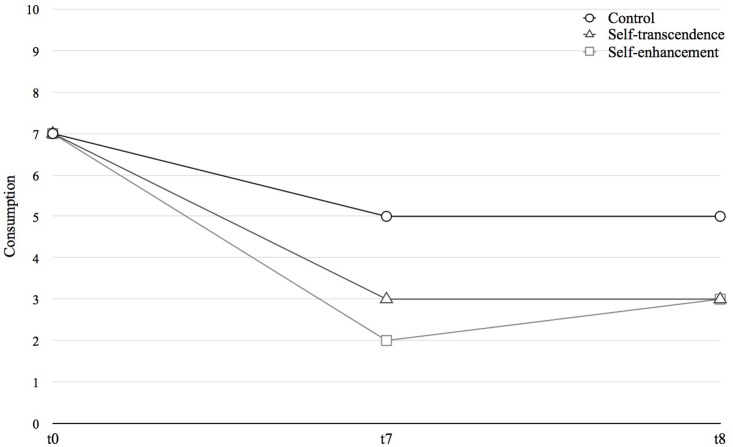
Proposed pattern of results showing reduced consumption within experimental groups.

To examine whether user-generated content will reduce excessive consumption, a multiple regression analysis will be calculated, using condition as an independent variable to predict excessive consumption post-intervention, taking the baseline measurement into account as a covariate, in addition to other controlled variables (e.g., base-line values, demographics). As can be seen in Figure 2, we expect a main effect of point of time for all conditions. In other words, we expect to see consumption behavior decreased post-intervention. Additionally, we expect to see an interaction between point of time and condition, showing a stronger reduction of excessive consumption in the experimental groups following the user-generated content intervention in comparison to the control group.

The strength of the effect is expected to be higher between t0 and t7 than between t0 and t_end_. In other words, we expect a stronger effect during the intervention compared to the post-intervention phase. Condition will be contrast coded to facilitate the interpretation of the interaction (Cohen et al., 2013). This analysis will also be used to examine which aspects of user-generated content affect excessive consumption, comparing self-transcendence to self-enhancement conditions. Although, there is no clear expectation on which content will have a stronger effect, this will be examined through exploratory analyses. Participants' in-take reported motivations, values, involvement, and personal goals to reduce consumption will be considered as control variables in these analyses.

### Consumption reduction-intent

A similar regression analysis will be calculated exploring the interventions' effects on reported intent to reduce consumption. Consumption reduction intent is expected to increase, especially for participants taking part in the interventions, showing both a main effect of point of time during the experiment, and an interaction effect between point of time and condition.

Furthermore, a correlational analysis, examining the link between the intent to reduce consumption and reported behavior, will be performed. Pearson's correlation coefficient will be calculated between reported behavior and reported intent, controlling for reported commitment to the study. We expect to find a negative link between consumption reduction-intent and consumption behavior.

### Effects of reduced consumption

Using another regression, we will further analyze questionnaire data collected at the beginning, during the intervention, as well as during the post-questionnaire, to investigate, whether reduced consumption affects life-satisfaction. We expect it to increase within both experimental groups, negatively correlating with excessive consumption.

### Changes in values and motivations

Using a repeated-measures ANOVA, with *post-hoc* contrasts, we will also compare in-take measurements of participants' values and motivations to the two post-intervention measurements. Thus, we will test whether exposure to the intervention stimuli affected consumer values and motivations directly. We expect participants in the self-transcendence condition to show an increase in biospheric values and motivations at t7, while participants in the self-enhancement condition will show an increase in egoistic values and egoistic and hedonic motivations at t7. Control condition participants are not expected to show a significant change in either values or motivations during the intervention. This measurement will serve as a manipulation-check. We will also explore whether the manipulation persists at t_end_, one month following the intervention.

## Anticipated results

We expect this project to provide insight into the effectiveness of user-generated content in reducing excessive consumption. We hope the project will shed light on specific aspects of user-generated content that help reduce excessive consumption and increase well-being. We predict to find data supporting our hypotheses and we therefore anticipate reduced excessive consumption within the intervention groups, as well as increased life-satisfaction.

Nevertheless, this research project entails some methodological challenges. One project challenge is the limited intervention time. A one-week intervention may not be sufficient time to produce significant impact on consumer intent and behavior. However, we chose this short time-period to minimize respondent's time-burden; prolonged intervention periods could increase potential issues in participant commitment and dropout rates.

Additionally, our choice of a control group is limiting. Control groups in intervention studies could be designed in several ways, and they should provide a comparison measurement to that of the intervention. Our findings will be limited in that they do not control for other, non-video interventions, such as written-text or monetary-incentive interventions. By pinging participants daily, and asking them to answer a mood scale, our outlined control condition does control for a placebo effect and a priming effect. Since our research question is inspired by a phenomenon currently occurring in the field (i.e., YouTube Minimalism videos) our control condition allows us to test whether these videos are more beneficial for consumers than having no engagement in a consumption-reduction intervention. Thus, the control condition chosen for this study is not optimal, but it does produce the highest ecological validity. If feasible, both monetarily and logistically, we recommend authors utilizing this protocol include additional control conditions to their design.

Due to the exploratory nature of the study, it is possible, that neither intervention significantly impacts buying intent, behavior, affective state, or life satisfaction. Nevertheless, null results will be highly informative. As evident in the number of Minimalism videos on YouTube, and number of subscribers to this content, it would be expected that this user-generated content is effective in promoting voluntary simplicity. If the outlined study finds that even in controlled settings there is no support for a significant influence of this content on behavior, our results could improve the public's understanding of user-generated content. The results—whether supporting the hypothesis or not—will raise further scientific questions and influence our handling of user-generated content.

In sum, the proposed project aims to contribute to the limited literature on voluntary simplicity and the use of user-generated content in consumer interventions. Specifically, this study might add a literary contribution to consumer values and sustainable behavior, specifically by examining its applicability in promoting voluntary simplicity and reducing excessive consumption through media primed with self-transcendence or self-enhancement messages. Applied implications of the proposed study will be directed to social marketers, social media experts, and proponents of voluntary simplicity.

## Ethics statement

This study has received ethical approval from the Cambridge Psychology Research Ethics Committee.

## Author contributions

This study was conceived by AH. All of the authors (AH, AB, JB, ND, MF, KS, and FS) contributed to the research design, method, analysis plan, and potential implications discussion. All authors approved the final manuscript.

### Conflict of interest statement

The authors declare that the research was conducted in the absence of any commercial or financial relationships that could be construed as a potential conflict of interest. The reviewer EP and handling Editor declared their shared affiliation, and the handling Editor states that the process nevertheless met the standards of a fair and objective review.

## Footnotes

^1^See for example; https://www.youtube.com/watch?v=AhJc8wtQbxQ (YouTube-video), https://www.reddit.com/r/minimalism/comments/53l2m3/extreme_minimalists_post_your_storystuff_here/ (online forum), http://time.com/3738202/minimalism-clutter-too-much-stuff/ (news article), http://www.theminimalists.com/minimalism/ (website), http://minimalismfilm.com/ (documentary).

^2^Addiction Resources: http://www.nhs.uk/Livewell/addiction/Pages/addictionwhatisit.aspx
