# Supplementary material for: A Study Protocol for Testing the Effectiveness of User-Generated Content in Reducing Excessive Consumption
Source: Front Psychol. 2017 Jun 9;8:972. doi: 10.3389/fpsyg.2017.00972 (PMC5465384; doi:10.3389/fpsyg.2017.00972)
Supplement: Supplementary file 1 [file Table1.docx]

**Appendix A: Video check-list example**

*Video 1: What is Minimalism?*

| **Topic** | **Self-transcendence** |  | **Self enhancement** |
| --- | --- | --- | --- |
| Content | Introduction to Minimalism | | |
|  |  |  |  |
| Condition Introduction | *“Welcome to the first video in the Minimalism series. In this video, I will discuss how I was introduced to Minimalism and what this lifestyle entails.*  *“We are here because we want to* ***make the world a better place.*** *By being* ***environmentally friendly, conscience*** *and* ***ethical****, we can* ***make a positive impact on the world***.” |  | *“Welcome to the first video in the Minimalism series. In this video, I will discuss how I was introduced to Minimalism and what this lifestyle entails.*  *“We are here because we want to* ***make our lives better.*** *By* ***being less stressed,*** ***more in control****, and* ***spending wisely****, we can* ***make a positive impact on our own lives***.” |
| Opening | *“This vlog consists of different episodes and I will focus on different topics in each one. I will share my story about how I started living as a minimalist and how I managed to declutter. Then, I will talk about how I managed to reduce my consumption, and how I faced obstacles along the way. In order to give you some insight into how it feels and what it really takes to adopt Minimalism, I will share my personal stories with you. I will also point out some specific, realistic goals that you could set for yourself, and I will suggest some methods you could implement in order to achieve these goals.”* | | |
|  |  |  |  |
| Gratitude for watching | *“I’m glad you are interested in watching my videos about Minimalism. I would love to give you a quick introduction into what ‘Minimalism’ is and what it means to me. I will also provide you with some of my own personal experiences and tips.”* | | |
|  |  |  |  |
| Definition | *“Minimalism means realizing that you can live by positive values with fewer clothes, fewer furniture, fewer interior design things. Simply fewer material items. For me, being a minimalist means realizing that I am true to my values when I reduce my material consumption.”* | | |
|  |  |  |  |
| Project 333 | *“There is this famous project that I personally adopted called Project 333. This project challenged me to only use 33 pieces of clothing for 3 months. It was during those months that I realized how good it was to get rid of so many items and how great it was to simplify my life. I felt that I was being true to my values in a way.”* | | |
|  |  |  |  |
| Condition closing | *“In my vlog I would like to show you how you can set your goals to get started and steer your life in the right direction.*  *“I will help you get to know Minimalism and learn about* ***the environmental goals you can achieve****.”* |  | *“In my vlog I would like to show you how you can set your goals to get started and steer your life in the right direction.*  *“I will help you get to know Minimalism and learn about* ***the well-being goals you can achieve****.”* |
| Style | *A didactic style of video, relatively neutral in tone. Visual stimuli will be a frontal shot of the presenter, sitting in a visually clean room. Camera focus above the bust. Audio clips accompany opening and closing of the video only.* | | |

**Appendix B: Non-validated questionnaires; Non-essential expenditures & Shopping frequency**

**SECTION A: Average weekly non-essential expenditures**

Non-essential items and services can be defined as things irrelevant to the chief purpose of your life. This can be an excess of, for example, clothes, electronics, snacks and coffee drinks, makeup, etc. that you don’t need to live your everyday life.

Question 1: What non-essential item/service, in your opinion, do you

spend too much of your money on?

Response: _____________________________________

Question 2: How much money *on average* did you spend last week on [response to

question 1] in general?

Response (amount and currency):______________

Question 3: In *your personal opinion*, how much in general did you spend last week on

[response to question 1]?


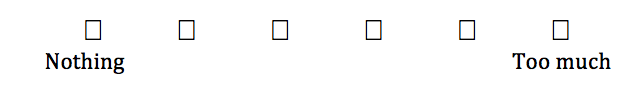


Question 4: How much *on average* did you spend last week on non-essential items in general?

Response (amount and currency):______________

Question 5: In *your personal opinion*, how much in general did you spend last week on

non-essential items?


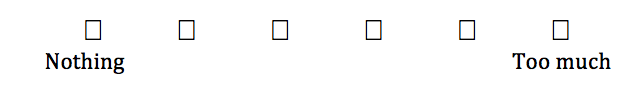


Question 6: If you find a good deal on something you don’t really need during a shopping trip, how likely are you to buy it anyway?


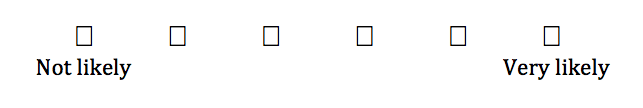


Question 7: How likely are you to buy something you don’t need in the upcoming week?


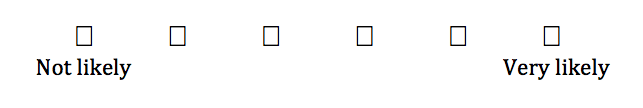


**SECTION B: Shopping frequency**

Shopping means different things to different people. When we refer to shopping, we mean shopping for non-essential things. So, “going shopping” doesn’t include, for example, buying food at the supermarket or getting gas for your car. Going shopping can also be done at home, online, and does not require that you actually go out. Lastly, going shopping doesn’t require that you buy something—you should have an intent to buy something, but might end up only browsing.

Going shopping, for example, could be done by browsing stores’ websites for new electronic devices, or visiting a retail clothing store at a mall.

Question 1: How often, in your opinion, do you typically go shopping in a week?


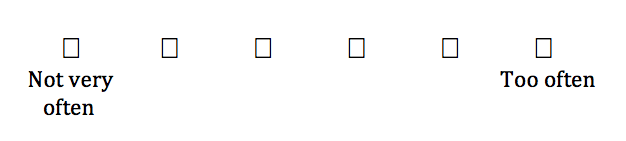


Question 2: How many times do you typically go shopping in a week?

Response:___________________
